# Using an Individual-Centered Approach to Gain Insights From Wearable Data in the Quantified Flu Platform: Netnography Study

**DOI:** 10.2196/28116

**Published:** 2021-09-10

**Authors:** Bastian Greshake Tzovaras, Enric Senabre Hidalgo, Karolina Alexiou, Lukaz Baldy, Basile Morane, Ilona Bussod, Melvin Fribourg, Katarzyna Wac, Gary Wolf, Mad Ball

**Affiliations:** 1 Center for Research & Interdisciplinarity INSERM U1284 Université de Paris Paris France; 2 Open Humans Foundation Sanford, NC United States; 3 École centrale d'électronique Paris France; 4 Center for Research & Interdisciplinarity Paris France; 5 Quality of Life Technologies GSEM/CUI University of Geneva Geneva Switzerland; 6 Article 27 Foundation Berkeley, CA United States

**Keywords:** symptom tracking, COVID-19, wearable devices, self-tracking, citizen science, netnographic analysis, cocreation

## Abstract

**Background:**

Wearables have been used widely for monitoring health in general, and recent research results show that they can be used to predict infections based on physiological symptoms. To date, evidence has been generated in large, population-based settings. In contrast, the Quantified Self and Personal Science communities are composed of people who are interested in learning about themselves individually by using their own data, which are often gathered via wearable devices.

**Objective:**

This study aims to explore how a cocreation process involving a heterogeneous community of personal science practitioners can develop a collective self-tracking system for monitoring symptoms of infection alongside wearable sensor data.

**Methods:**

We engaged in a cocreation and design process with an existing community of personal science practitioners to jointly develop a working prototype of a web-based tool for symptom tracking. In addition to the iterative creation of the prototype (started on March 16, 2020), we performed a netnographic analysis to investigate the process of how this prototype was created in a decentralized and iterative fashion.

**Results:**

The Quantified Flu prototype allowed users to perform daily symptom reporting and was capable of presenting symptom reports on a timeline together with resting heart rates, body temperature data, and respiratory rates measured by wearable devices. We observed a high level of engagement; over half of the users (52/92, 56%) who engaged in symptom tracking became regular users and reported over 3 months of data each. Furthermore, our netnographic analysis highlighted how the current Quantified Flu prototype was a result of an iterative and continuous cocreation process in which new prototype releases sparked further discussions of features and vice versa.

**Conclusions:**

As shown by the high level of user engagement and iterative development process, an open cocreation process can be successfully used to develop a tool that is tailored to individual needs, thereby decreasing dropout rates.

## Introduction

### Background

Patient- or participant-led research has been suggested to improve self-management capabilities [1] and provide ways to generate otherwise undone science [2,3]. A particular subtype of participant-led research is personal science, which involves the use of empirical methods by individuals to pursue personal health questions [4]. Personal science is a distinct category of citizen science that has emerged from the Quantified Self community and its efforts to advance participant-led research [5,6]. In personal science, practitioners almost always take the lead in all stages of the research process by definition [4]. Owing to this high level of individual engagement and tailoring to individuals’ interests, personal science has the potential to deliver novel insights relevant to its practitioners [7], which can lead to an improved sense of agency and quality of life [8]. Furthermore, the insights and self-expertise generated by these types of participant-led processes have potential relevance for professional and scientific research, both topically as a source of ideas and methodologically as a source of tools, analytical approaches, and workflows [9].

Wearable devices—from wristbands to smartwatches and other personalized, miniaturized on- and around-body devices—are frequently used by self-trackers. These devices are becoming increasingly common and are used for a wide spectrum of well-being, fitness, and health-related purposes [10]. This is further facilitated by the fact that the number of sensors used in these devices is growing rapidly. In addition to accelerometers and gyroscopes to track physical activity, sensors to measure physiological signals such as heart rate, body temperature, respiratory rate, and blood oxygen saturation, which may correspond to the health or sickness state of the human body [11], are also frequently found in wearables [12,13]. Consequently, even outside the realms of personal science, wearables have long been seen as promising tools for facilitating health-related monitoring and enabling personalized medicine [14,15] and have been proposed or used to monitor conditions as diverse as cardiovascular disease [16,17], Alzheimer [18], and graft-versus-host disease [19].

In response to the COVID-19 pandemic, interest in using wearable technology for infection prediction and surveillance has increased [20-22]. Anecdotal reports from self-trackers suggest that wearables may provide evidence of COVID-19 infection [23]. During the first year of the COVID-19 pandemic, a small number of studies appeared, highlighting that wearable devices, often along with self-reported symptoms, might indeed be used for the early detection of COVID-19 infections and to assess physiological symptoms [24-27]. The majority of these studies take a crowdsourcing-based approach—in which participants are invited to contribute by providing their own wearable data along with regular symptom reports and COVID-19 test results—as the main way of engaging individuals. The goal of the data collection process in these studies is to create big data sets to interrogate.

In contrast, there have been limited efforts to engage personal scientists in cocreating such symptom tracking efforts. Personal science practices are largely done in isolation, and the Quantified Self movement has consequently accumulated limited knowledge so far [8]. To fill this gap, we present a case study of Quantified Flu (QF), a project cocreated by a community of personal science practitioners in response to the COVID-19 pandemic.

### Objective

The goals of this work are twofold. First, we documented the contrasting cocreation approach of QF with its focus on personal science rather than large-scale research. To this end, we used netnographic methods to document how the cocreation process developed and generated a citizen science platform prototype over a relatively short period. Second, we explored the consequences of the projects’ contrasting cocreation approach and focused on personal science, particularly with respect to the ultimate design of the QF tool and its use.

## Methods

### Overview

The cocreation process of this study is based on an action research approach [28], simultaneously for developing a useful community resource while also generating shared knowledge about the process. In our case, action research was implemented through practical work to support the participatory design of a digital platform [29,30] under open-source principles [31], followed by netnographic data collection and analysis to understand its development and usefulness as a cocreation process [32]. For this, all authors except ESH were involved as participants in the cocreation process, in collaboration with the rest of the participants, during the iterative prototyping of the QF platform.

### Community Cocreation Process

QF began with a discussion on the monthly Open Humans (OH) community call at the beginning of the COVID-19 pandemic on March 10, 2020. OH is a platform for empowering individuals around their personal data, to explore and share research processes for the purposes of education, health, and science in general [33]. The community calls involved 83 individuals so far (until September 3, 2020), and the monthly calls are frequented by a mix of citizen science and personal science practitioners; usually, around 10 individuals take part in each call. Following an initial brainstorming, the discussions and planning stages were continued through the following community calls and a dedicated communication channel of the OH community Slack [34]. Furthermore, over the evolution of the project, other communities such as Quantified Self [35] and OpenCovid19 Initiative [36] were engaged and involved in different aspects of the development of the project.

In parallel to 10 additional community calls between March 10 and September 3, 2020, the main coordination tool for the QF project was a specific Slack channel, with a total of 146 subscribers and 34.2% (50/146) active users over time with different levels of involvement and activity. During this timeframe, this openly accessible channel gathered a total of 844 messages from these users, with a total count of 26,691 words (and 3917 unique words).

Although the planning, coordination, and social aspects of the cocreation process mainly took place on the mentioned project’s Slack channel, technical collaboration and software development occurred through GitHub and the git repository of the QF. Due to the iterative nature of open-source collaboration, no up-front requirement analysis was performed. Instead, prototypes were developed over time according to community discussions by iteratively adding and testing implementations. On GitHub, 7 contributors created a total of 316 commits since March 12, 2020, leading to the technical prototype outlined later. The source code for the project is available under an open license on GitHub [37].

### Netnographic Content Analysis

To investigate and analyze the cocreation stages that led to the QF prototype, we performed a netnographic analysis of its iterative communication process, similar to previous studies on cocreation in health-related community settings [38]. Netnography is an interpretive research method derived from ethnography, usually applied to social interaction processes in digital channels and platforms, and focused on digital traces of public conversations as analyzable data. As a qualitative technique broadly applied to the study of web-based communities [32], Netnography allows capturing and reflecting interactions as an observational, inductive, and unobtrusive approach while combining it with participatory methods [39]. In particular, we examine how individuals engaged in the QF Slack channel for the collaborative development of the QF platform as a case study setting [40].

For this part of data collection, one of the researchers (ESH) developed a codebook combining key concepts of cocreation and collaboration in communities of practice (Textbox 1). The codebook was cross-checked for validity by 2 other authors (BGT and MB). Following this, it was applied to the QF Slack channel posthoc without this specific researcher (ESH) having participated in the previous community discussions.

**Codebook for Quantified Flu Slack communication message content analysis.**

**Communities of practice-related messages**

- Socialization
  Support or coordination: parallel messages regarding overall coordination and personal and empathic support interventions
  Possible collaborations: ideas regarding potential collaborators and connections to other organizations or experts who can support or contribute to the project
  Outreach: messages related to the visibility of the project, possible dissemination, or alliances for spreading the process
  Off topic: nonrelated messages to any of the previous (eg, about personal issues or intention-to-buy wearables)

**Cocreation-related messages**

- Ideas
  Inspiring or similar initiatives: mentions to other COVID-19-related projects being developed or known externally
  COVID-19 related: links to news or updates regarding the COVID-19 pandemic and its evolution
  Mention to tool or wearable: references to a specific wearable for its potential connection to the Quantified Flu project
  Scientific knowledge or papers: mentions or links to studies or publications and elaborated scientific knowledge
- The Quantified Flu concept
  Goal setting or discussion: concept-related interventions about the objectives of the project
  Protocol or tool design: mentions to how the protocol and tool should work or specific aspects of its possible design
  Feature suggestion: interventions suggesting specific characteristics or new possible features of the tool
  Pattern or data observation: statements regarding the observation of data in relation to the goals or possible functioning of the project
- The Quantified Flu prototype
  Incremental development or updates: messages informing about new implementations and code development of features applied to the prototype
  Technical issues: specific technical issues to solve or observations about needed improvements for correct use
  Help testing: interventions asking or offering support in testing the tool by community members
  Help developing: interventions asking or offering technical support for the development of the tool

This part of data analysis was used to determine the typology of messages regarding the cocreation of the QF platform, from idea to concept to prototype [29], and other types of messages relevant from a communicational and empathy-needed dialogic process in communities of practice [41]. Each Slack message was assigned up to three top tags based on the aforementioned codebook categories, depending on its text density and characteristics. The researcher (ESH) assessments of types and categories of messages were subsequently reviewed and discussed by another coauthor (BGT), who was actively involved in the analyzed cocreation process.

## Results

We present the current prototype of the QF platform [42], as a result of the described technical development, before analyzing the cocreation process that led to it.

### Community-Based Development

The first overview derived from our netnographic analysis of the four main categories of messages interchanged during the cocreation of the QF prototypes on its dedicated Slack channel (March 10 to September 3, 2020) shows a relative balance in the topics of the web-based messages among the 1171 message fragments that were annotated (Textbox 2).

**Figure 1:**
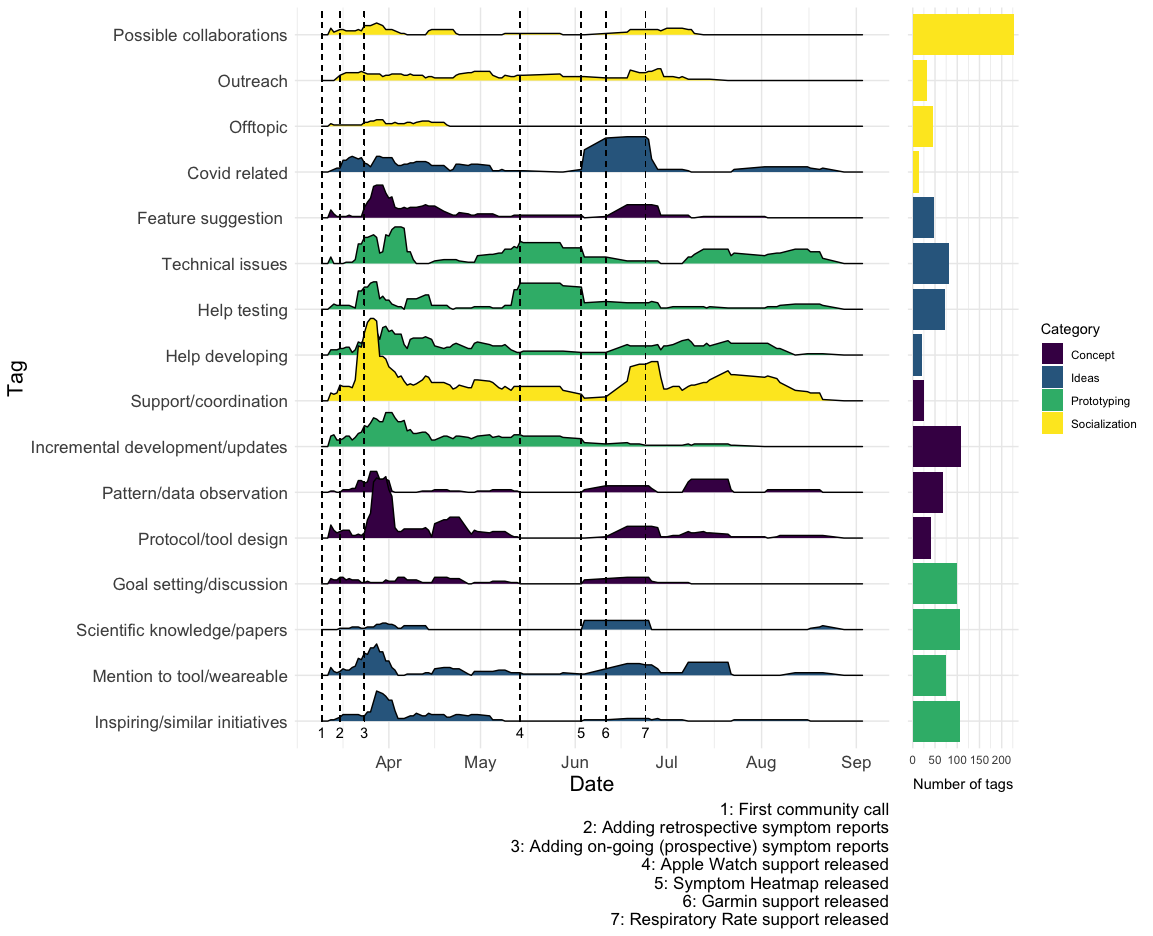
Distribution of message types over time; the frequency of tags are given as 7-day rolling averages. Events 1-7 around Quantified Flu development are given as vertical lines. Bar plots show the total number of tags per category.

Overall, during the development of QF, the *Prototyping* and *Socialization* messages were slightly more common than the *Concept* and *Ideas* ones (Figure 1). On the level of the tags or subcategories, the most frequent ones are *Support or coordination* (227), *Protocol or tool design* (109), *Technical issues* (107), and *Help developing* (106).

Focusing on these more specific tags, as defined in the codebook (Textbox 1), within each category over time (Figure 1), we observe that all the four main categories, as well as the individual tags, are present over the whole time frame of cocreation from early April to September 2020. In particular, messages regarding *Support or coordination* are present throughout the entire time range. Other recurrent message types during the analyzed time span fall within the categories *Ideas*, *Concept,* and *Prototyping*, highlighting the iterative design, implementation, and testing participatory processes that took place to develop and improve the QF prototype over time.

Importantly, the *Protocol or tool design*, *Mention to tool or wearable*, and *Feature suggestions* categories, which are indicative of the cocreation process, appear early on but remain active in bursts throughout the full observed time span, often following new releases of the QF prototype. In addition, the *Help developing* and *Help testing* categories remain active over the whole duration of the prototype development, with the former showing a more constant activity (mean 1.1 tags per day, SD 1.9) whereas the latter appears in bursts (mean 0.76 tags per day, SD 2.1) around new feature releases.

**Examples of messages that were tagged according to the codebook used for the netnographic analysis.**

**Socialization**

- Support or coordination
  “Very good community call focused on quantified flu this morning. Glad I participated”
  “Great updates, thank you and I am definitely staying tuned...how can I help other than visualisations. Have a good rest of the day!”
- Possible collaborations
  “[Person outside the community] is usually sitting in the office right next to mine (working from home these days) and we’ll do a call tomorrow to chat about synergies!”
  “It’s possible we could address these with support from the company, which in turn depends on our convincing them to prioritize this support. We have some close contacts there that could lead to success”
- Outreach
  “We’re (very briefly) featured in the latest UCSD newsletter”
  “Oh, and we already got some media coverage in the german ‘digital living’ magazine t3n”
- Off topic
  “Not sure if it’s appropriate to ask but does anyone have a way to get discount on the Oura ring?”
  “BTW, semi-related to this project: Just coinciding with the general lockdown in Paris I stopped smoking and could nicely see my resting heart rate drop, my heart rate variability grow, etc. within the first few days”

**Ideas**

- Inspiring or similar initiatives
  “Another flu-tracking app, from Duke:...[URL]”
  “Looks like Michael Snyder’s famous self-tracking lab at Stanford is doing something similar”
- COVID-19 related
  “Placing this link here because it was an interesting symptom diary someone shared on Twitter they made”
  “Btw. during the community call yesterday, [community member] shared this symptom report of a contributor who thinks he has covid19”
- Mention to tool or wearable
  “How far is the Fitbit Intraday integration? As of right now the Fitbit Graph seems quite a bit less detailed than the Oura one”
  “The Garmin devices are a bit tricky, as their API is locked off unless you apply for access with them”
- Scientific knowledge or papers
  “August 31 Webinar from hlth.com: Wearable Technology’s Potential to Help Detect Illness”
  “Stanford’s 2017 paper was all about longitudinal health data and health outcomes”

**The Quantified Flu concept**

- Goal setting or discussion
  “Personally, I see the purpose of this project not so much as epidemiological, but about expanding the personal value of our data. Doing that as a group helps us learn from context, as well as individually”
  “This project might also be a starting point for prospective tracking for people that get sick, going forward. Still thinking about if/how that would work.”
- Protocol or tool design
  “So i think it may make sense to give numerical values for each symptom, from 1-5 or 1-10 in terms of intensity, and also timestamp them to allow for multiple logging within a day”
  “but my vicks smart temp thermometer arrived...the associated app allows me to record...medication, symptoms (cough, sore throat, chills, body ache, ear ache, nausea, stomach ache, fatigue, short breath, headache, diarrhea, runny nose), a free text ‘notes’”
- Feature suggestion
  “She wears an Apple Watch and has resting heart rate data. Should I invite her join quantified flu even though she does not have an Oura or Fitbit? Is adding support for Apple Watch too much work at this stage? Manual entry wouldn’t be very challenging probably”
  “I’m realizing the public list could at least give event IDs so you have some sort of identifier for each one”
- Pattern or data observation
  “Already contributed two sick events of mine from 2019, that are very obvious in the data but also quite different”
  “So I don’t think it’s necessarily measurement noise. For my own data my gut feeling is that all variations ≤ ±0.3 °C are probably just daily fluctuations for a myriad reasons”

**The Quantified Flu prototype**

- Incremental development or updates
  “Some publicly available data now – you can explore on the site, and there’s JSON endpoints to get raw data”
  “Hey <!channel>, we have another nice visualization update thanks to [community member]! The retrospective events now have the same display that can be found for the ongoing symptom reports, check out [QF link] for an example!”
- Technical issues
  “Oh, not sure if that’s true though! I think if the oura dies while doing the recording it doesn’t deliver any data (happened to me 3-4 times with my broken oura where it would not record anything for the night)”
  “Also, I found a strange inconsistency in the data. For one of the users, the JSON file states that they are sick on July 11th, but the interactive display on the website does not (the JSON says that the person had a sore throat, but the web display does not). I attach the examples”
- Help testing
  “My daily symptom checkins have stopped, is this happening for anybody else? I thought it might be an email issue on my side”
  “Does anyone of you have an android watch/wearable that would track heart rate to test whether it works?”
- Help developing
  “Hi everyone! I am a programmer and would be happy to help. I have lots of experience with python”
  “I thought a cool starting visualization could be a heatmap similar to the github activity view, but with time only on the x-axis and the different symptoms on the y-axis and colored by symptom severity. If you have other cool ideas for appealing and insightful visualizations feel free to let us know!”

### QF: Technical Platform Implementation

#### Overview

**Figure 2:**
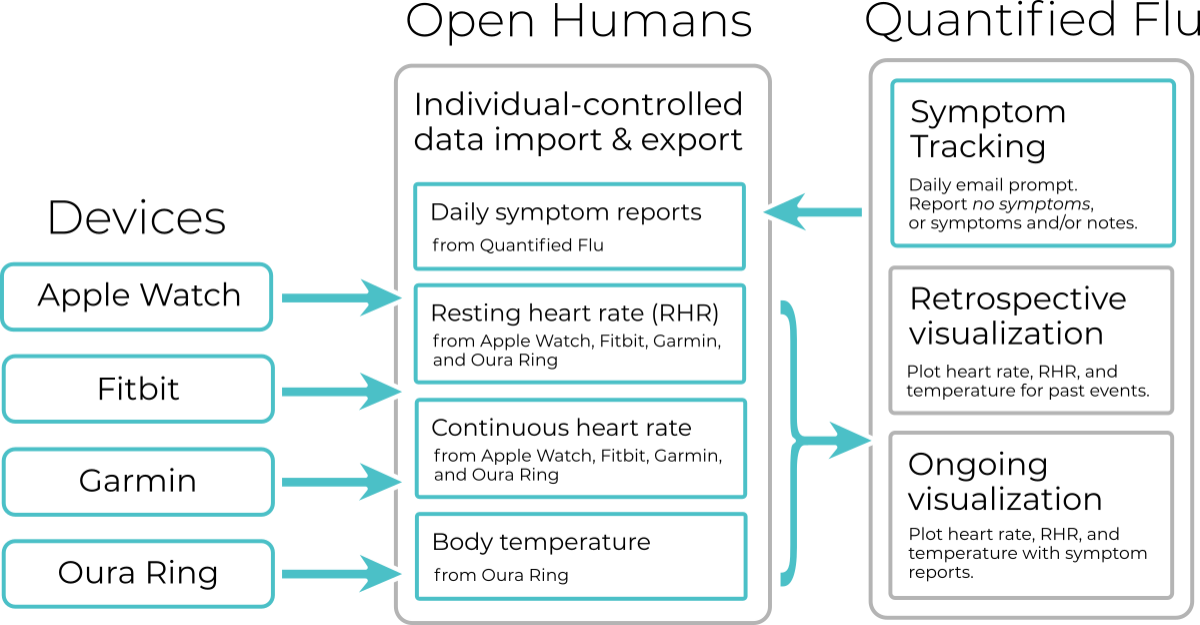
Data flow and user flow in Quantified Flu. RHR: resting heart rate.

As a result of this community-based development process, QF evolved into a responsive web application that can connect to a wide variety of devices, implemented in Python or Django programming language. Users must be registered on OH, having the option of linking a range of available wearable devices from which physiological data (heart rate, body temperature, and respiratory rate) can be imported into the OH platform; visualize past sickness or infection events (retrospectively) on it (present since the first prototype, launched on March 16, 2020); and engage in daily (prospective) symptom tracking (added in the second prototype, released on March 24, 2020; Figure 2).

#### User Accounts, Data Storing, and Anonymization

To enable rapid prototyping, QF is connected to the OH platform [33] as a back end to manage user permissions and store user data. OH provides OAuth2-based application programming interfaces (APIs) to authenticate users while keeping each user pseudonymous to the QF platform, as no personally identifiable information is transmitted. Instead, only a random 8-digit user identifier specific to the QF project is provided. Furthermore, OH provides APIs to access and store user data in their system through those identifiers and provides methods for users to consent to share data from the OH platform with third parties such as QF.

#### Wearables

To further bootstrap the creation of the prototype, QF made use of the existing wearable integrations that OH already offered (Fitbit daily summaries, Fitbit intraday data resolution, and Oura Ring). To facilitate usability, QF also integrated these data import methods directly into the prototype, using OH as the data store for the wearable data.

Furthermore, following community suggestions and ideation discussions (Textbox 2; *Mention of tool or wearable*), QF also added Google Fit (May 6, 2020), Garmin (June 11, 2020), and Apple Health (May 14, 2020) as additional supported wearable devices. Depending on the wearables, users can import and use their heart rate throughout the day, daily resting heart rate, body temperature, and respiratory rate in QF (Table 1).

**Table 1:** Wearables supported by Quantified Flu.

Wearable	Development	Resting heart rate	Heart rate throughout day	Body temperature	Respiratory rate
Fitbit	Existing	✓^a^			
Fitbit Intraday	Existing	✓	✓		
Oura Ring	Existing	✓		✓	✓
Google Fit	Extended (added heart rate data)	✓			
Apple Health	Added	✓	✓		
Garmin	Added	✓	✓		

^a^The feature is measured by the wearable.

Unlike other wearables integrated into QF, Apple Watch does not provide a web-based API to access and export data. Thus, following another community suggestion (Textbox 2; *Feature suggestion*), a mobile iOS app was created to provide a link to QF. This specific app enables users to export their heart rate data collected by Apple Watch. The source code for this mobile app is also available under an open license [43].

#### Symptom Tracking

Users can report symptoms using a QF website. On the basis of previous works [24-26] and early community discussion and feedback (Textbox 2; *Protocol or tool design*), QF implemented a list of 12 symptoms that were classified as respiratory, gastrointestinal, and systemic symptoms (Textbox 3), allowing users to score those on a 5-point scale (1=light; 5=worst). In addition, users can report fever measurements and use free-text fields for the suspected origin of their symptoms, further symptoms, or notes to put their symptoms into context (Textbox 2; *Protocol or tool design*).

**Symptoms of sickness that users can monitor in Quantified Flu.**

**Respiratory**

- Cough
- Cough with mucus or phlegm
- Reduced sense of smell or anosmia
- Runny or stuffy nose
- Sore throat
- Shortness of breath

**Gastrointestinal**

- Diarrhea
- Nausea or vomiting

**Systemic**

- Chills and sweats
- Fatigue and malaise
- Headache
- Muscle pains and body aches

Users can opt in to receive daily symptom report reminders that are sent through the anonymous OH email system at a user-selected time, as another tool feature that was discussed and regularly tested by participants (Textbox 2; *Help testing*). Each email contains the following two links: (1) the *reporting no symptoms* link, a single-click link that requires no further interaction of the user, and (2) the *reporting symptoms* link, which takes users to the symptom report form.

#### Data Visualization

##### Overview

To provide users with easy ways to facilitate understanding of their own physiological data and potentially explore it in relation to their own symptom reports, QF used *D3.js* to create interactive visualizations. These visualizations present the evolution of the various physiological data points and put them into the context of their symptom reports where available.

The QF platform provides personal science practitioners 2 main ways to explore their physiological wearable data in relation to infections—the retrospective analysis of prior events and an ongoing (prospective) analysis of symptom reporting.

##### Retrospective Analysis

**Figure 3:**
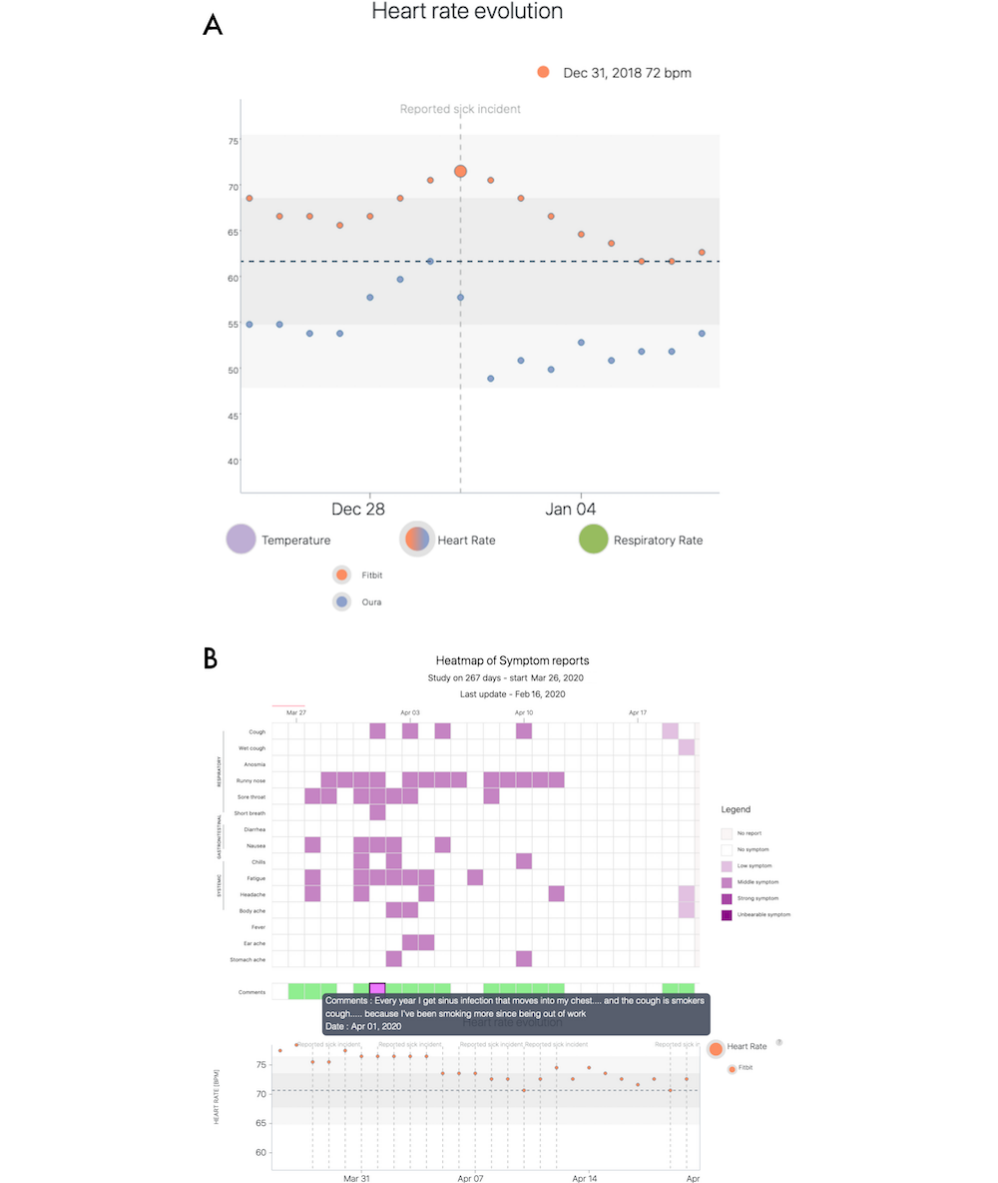
Screenshots of the Quantified Flu prototype showing typical visualizations generated by users. (A) An example data visualization of an individual, retrospective sickness incident that happened on December 31, 2018. The data plotted are the resting heart rate recordings as measured by the Fitbit and Oura Ring. (B) An example of an ongoing symptom report visualization. The top half shows a heat map of the symptoms that were present along with their strength, and green boxes display user-provided free-text comments. The bottom half shows physiological data from wearables. bpm: beats per minute.

Users can select a given, historic date on which they fell sick or had specific symptoms, and QF will, if available, extract wearable data for the 3 weeks before that date and 2 weeks after the incident. This allows users to visualize sickness incidents that occurred before the launch of QF. Depending on the wearable (Table 1), users are given the option to display different physiological variables over that 5-week time period and explore how they change over time. To facilitate the interpretation of changes and outliers in the graphs, both the first and second SDs are presented as well (Figure 3). Although users can add comments to retrospective events, detailed symptom reports are absent in this mode, as most users do not have detailed records of the historic sickness events. The retrospective analyses were part of the first prototype of the QF, launched on March 16, 2020.

##### Ongoing Symptom Reporting

Users can also report currently experienced symptoms through QF at any moment in time by selecting symptoms and their experienced strengths from a list (Textbox 3). This self-report is likely triggered by email, as explained earlier. Following symptom reports, users are automatically taken for their data visualization (Figure 3). On a wearable device data level, this visualization provides the same details as that of the retrospective analyses (*Retrospective Analysis* section). The ongoing symptom reports (Figure 3) were launched as a new feature in the second iteration of the prototype on March 24, 2020, also following discussion and contributions from the community (Textbox 2; *Incremental development or updates*).

In addition, this latter view aligns a heat map of each daily symptom report to the wearable data timeline, allowing the identification of patterns within the reported symptoms themselves and for visual cross-comparisons between the physiological data and the symptom reports. Furthermore, users can also access their comments for each symptom report from this visualization, allowing them to understand the contexts in which they made those reports.

### Community Use

A total of 190 personal science practitioners engaged with QF between its launch on March 16, 2020, and December 22, 2020. The initial prototype of QF (in place until March 24, 2020) only offered the possibility of analyzing retrospective sickness events. This feature was rarely used: only 24 users tried the feature, creating a total of 47 retrospective analyses. In total, 34 individual wearables were linked by these 24 users. The prospective ongoing symptom report feature was launched on March 24, 2020. In total, 92 users made use of this feature at least once, covering a range from a single symptom report being done up to over 300 reports for some members. Overall, 11,658 symptom reports were filed and 112 wearables were linked to it, between the launch of the feature and December 22, 2020.

**Figure 4:**
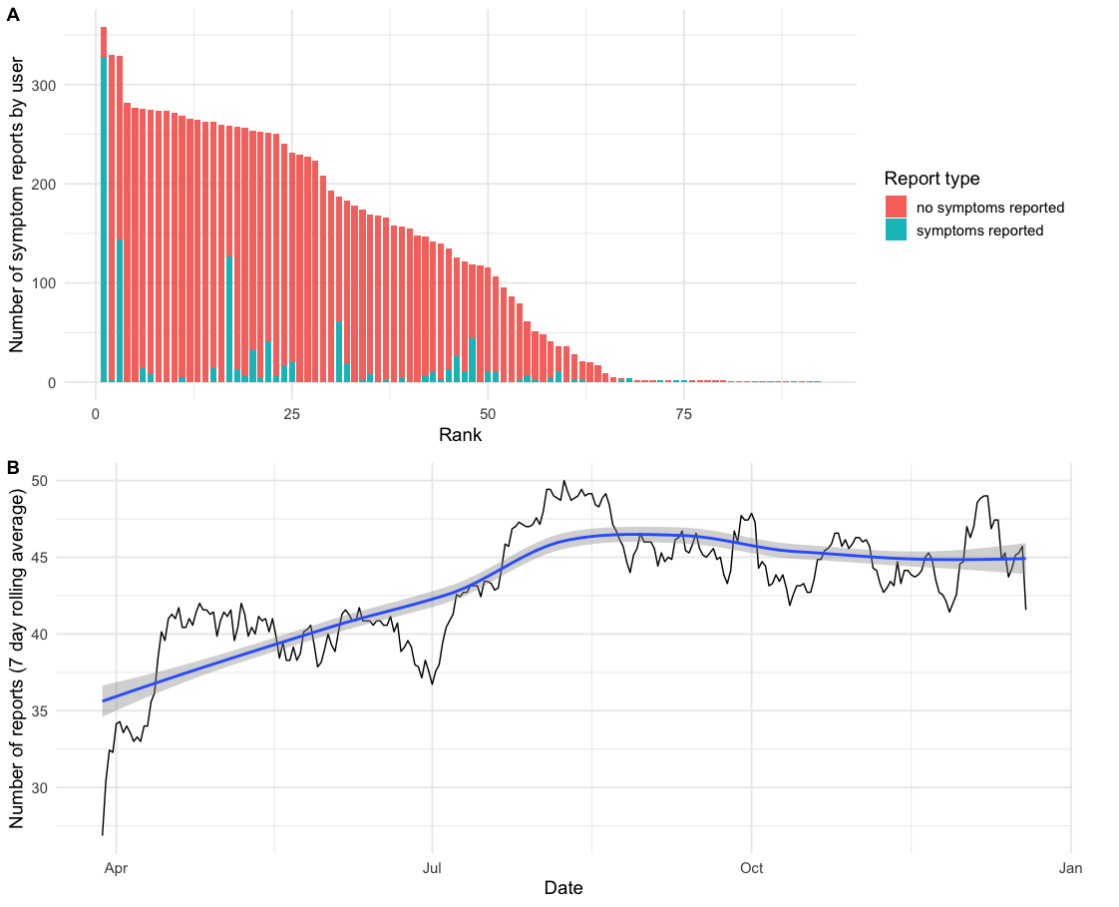
The use of Quantified Flu as measured by ongoing symptom reports filed by users. (A) Users were ranked by the number of symptom reports they have filed; data were broken down into whether symptoms were reported (blue) or not reported (red). (B) The number of symptom reports filed per day. Values were averaged into a weekly rolling average. The blue line represents the local regression–smoothed data along with SE (gray background).

The distribution of user engagement for the entire period (Figure 4), as measured by the number of reports, shows an approximately linear relationship between the number of reports done and the user’s rank of activity. The reports with symptoms are also not equally distributed across all 92 users, with a sizable fraction of users having no or only a few reports that include symptoms, whereas for some users, symptom reports comprise half or nearly all of the reports. Overall, the vast majority (10,594/11,658, 90.87% reports) were reports that included no symptoms. Of the 1064 reports with symptoms, 176 (16.54%) included explanatory notes or comments, in addition to the standardized symptom reports.

Looking at the number of symptom reports filed per day, we can observe a rapid rise in daily reports at the beginning of April 2020, reflecting the launch of the first prototype with the ongoing symptom reports. The second rise in daily reports started in July 2020, leading to the numbers starting to stabilize at around 45 symptom reports filed per day (by an average 45 users per day, SD 5; Figure 4).

## Discussion

### Principal Findings

In this paper, we present QF, a cocreated web-based project that enables personal science practitioners to engage with their own wearable data and visualize it in the context of when they are experiencing symptoms of potential infection. The spark that led to this community deciding to cocreate a symptom tracking tool was the beginning of the global COVID-19 pandemic, along with population-wide studies that made individuals wonder how useful their own wearable data might be for them in such a pandemic context. With its focus on individual learning, QF stands in contrast to various population-level studies performed to evaluate the usefulness of wearable technology for the prediction of illness [20,24-27]. At this individual scale, symptom tracking and health data more generally can offer support for individual sense-making on health experiences and conditions [44], which can be idiosyncratic and complex [45]. QF was also distinguished by a cocreation approach that targeted the individual learning and research interests of a web-based community and involved the iterative development of a digital tool in response to feedback, resulting in a format that attracted increased and sustained participation from early users.

One of the main aims of our work is to investigate the consequences of a cocreation approach that focuses on personal science: We observe that the initial QF prototype, which focused solely on retrospective symptom tracking, was rarely used. Only 24 users were engaged in this prototype. However, importantly, this initial version facilitated additional discussions about designing both the data collection protocol and extending the prototype (Figure 1), leading to the creation of the ongoing symptom reports as a feature launched in the next QF iteration. This feature received much more attention from the participants, with a total of 92 people using QF for their own regular symptom tracking, delivering some first insight into the importance and potential benefits of early engaging potential users in a health research design cocreation approach.

Furthermore, we observe that the level of engagement across these 92 QF users seems to drop linearly when ordered from most to least engaged users (Figure 4). This distribution is atypical for user engagement in web-based communities, where one typically observes power-law distributions for engagement [46]. Related to this, digital or mobile health apps in particular typically struggle with achieving continued use, as a large fraction of users drop out after a few interactions [47,48]. In previous studies, only 2% of initial users showed sustained use in the most extreme cases, with observational studies having an average dropout rate of 49% [49]. In contrast, around half of the QF users who engaged with ongoing symptom tracking did so on a regular basis, leading to 45 (SD 5) symptom reports per day on average (Figure 4), and over 50 users reported more than 3 months of symptom reports, highlighting continued longitudinal use. We argue that these uncharacteristically high numbers of user engagement, which is sustained over time, is a result of the community cocreation process that led to the final prototype of QF. Previous studies have found that users are more likely to continue using mobile health apps if there is a good fit between users and applications [50], which means that a cocreation process among future users could be a key way of achieving this fit.

For some users, this continued engagement might also be an indication that they experience regular or recurring symptoms, making them particularly interested in learning empirically about them through this specific kind of self-tracking. This is supported by the number of reports that include symptoms, where a subset of users reported having symptoms frequently, with some users reporting symptoms in 40%, or extreme cases even 90%, of the time (Figure 4). Further evidence for this comes from the notes or annotations that users can submit to the QF website along with their symptoms when filing their daily reports. Looking at the publicly shared notes in these reports, we find examples like “the cough is smokers cough...because I’ve been smoking more since being out of work” and “I was deep cleaning the house...all the dust got my allergies going again” highlighting possible reasons for recurring symptoms. Furthermore, these annotations help to provide context to individuals and others that aim to reuse publicly shared data. Although a severe case of coughing or nasal congestion might hint at acute infection, they might also be unrelated, as the annotations highlight. These contextual descriptions can be difficult to formalize, potentially explaining why symptom-based diagnoses are difficult to achieve in many cases [51,52].

Our second main goal, in parallel to the development of the QF prototype itself, is to explore how a community-driven initiative can contribute to collectively creating the tools needed to build self-knowledge by conducting a netnographic analysis of the main QF communication channel. Reflecting on the use of this qualitative and interpretative methodology for the study of web-based communities [53], we find that it adapts well to user-led prototypes, with some particular strengths and limitations. In the case of QF, we found that the netnographic approach was well suited to allow a posthoc study of the participatory design process after the prototype creation. This approach could be valuable in obtaining a better understanding of cocreation dynamics in similar health-related projects and studies [54], as it can be applied to existing text corpora of community interactions on digital text tools such as Slack, mailing lists, or forums. Its reliance on text communication is also one of the main limitations, as synchronous meetings—remote and physical—are less accessible as archival data, requiring recordings and transcriptions. Given this, it might be advisable to organize cocreation processes with Netnography techniques in mind to ensure adequately sized text corpora.

Applying such a netnographic approach to QF, we found a marked overlap of the various phases of ideation, conceptualization, and prototyping over time. Although a greater number of interactions can be found in the initial phases, there is a sustained regularity later on, particularly in areas such as feature suggestions or the design of the tool and protocol. In this sense, messages and interactions related to helping with development throughout the whole process reflect a typology of continuous and iterative cocreation, which is typical of collaboration processes in the development of open-source tools [55].

This iterative cocreation process is also highlighted in the burst-like appearance of feature suggestions and protocol or tool design discussions, which frequently appear following the release of new features, suggesting that new releases spark further protocol refinements and feature ideas, which in turn lead to the QF prototype refinement. Importantly, this means that the protocol itself, along with the concrete implementation, remains in a stage of flux over a longer period of time, compared with more traditional research design approaches. As a result, this type of collaborative approach is at odds with standard ethical oversight procedures for human subject research that require a precise predefinition of the protocol and the role of the individuals, whereas the main feature of cocreation is that it is emergent and adaptive, making detailed prespecifications impossible [56]. To fully take the advantage of the benefits of cocreation in the participant-led research, it might be necessary to develop different models of ethical oversight that recognize the autonomy of participants [57,58], to not discourage or stifle valuable forms of participant-led research [2].

Finally, it is also important to highlight how the other types of messages associated with communication in a community of practice context, which favor both web-based empathy and effective coordination, were produced in a prominent, constant, and sustained manner from the beginning of the cocreation process (*Support or coordination*; Figure 1). This mode of cocreation can be understood as an example of uninvited citizen science that relies on a shared set of values, self-stabilizing communication infrastructure, and a loosely defined coproduced knowledge object [59] (eg, the QF prototype itself). This way, the development of the data collection platform itself is framed in a dynamic, bottom-up, and adaptive way, similar to other open source and peer production experiences.

### Conclusions

Although QF is a project that is still at the prototype stage and with a correspondingly small user base, the cocreation processes of the platform prototype described here represent an example of how the codevelopment of digital research objects, within the relatively new participatory paradigm of extreme citizen science [60], can be implemented following bottom-up, dialogic approaches and a high level of participant engagement. This aligns with the still scarce literature on what has been called do-it-yourself science or peer-to-peer science [61,62], in which similar participatory approaches can offer an opportunity for early and sustained engagement from personal science practitioners in the collaborative definition of concepts, features, and protocols for health-related digital platforms.

## Abbreviations

API: application programming interface
OH: Open Humans
QF: Quantified Flu
